# Plasma proteomic data in bipolar II disorders and major depressive disorders

**DOI:** 10.1016/j.dib.2021.107495

**Published:** 2021-10-17

**Authors:** Hyunju Lee, Sang Jin Rhee, Jayoun Kim, Yunna Lee, Hyeyoung Kim, Junhee Lee, Kangeun Lee, Hyunsuk Shin, Hyeyoon Kim, Tae Young Lee, Minah Kim, Eun Young Kim, Se Hyun Kim, Yong Min Ahn, Jun Soo Kwon, Dohyun Han, Kyooseob Ha

**Affiliations:** a Department of Neuropsychiatry, Seoul National University Hospital, Seoul, Republic of Korea; b Department of Psychiatry and Behavioral Science, Seoul National University College of Medicine, Seoul, Republic of Korea; c Medical Research Collaborating Center, Seoul National University Hospital, Seoul, Republic of Korea; d Department of Neuropsychiatry, Kosin University Gospel Hospital, Busan, Republic of Korea; e Department of Psychiatry, Inha University Hospital, Incheon, Republic of Korea; f Proteomics Core Facility, Biomedical Research Institute, Seoul National University Hospital, Seoul, Republic of Korea; g Department of Pathology, Seoul National University College of Medicine, Seoul, Republic of Korea; h Department of Neuropsychiatry, Pusan National University Yangsan Hospital, Yangsan, Republic of Korea; i Mental Health Center, Seoul National University Health Care Center. Department of Human Systems Medicine, Seoul National University College of Medicine; j Seoul National University Medical Research Center, Institute of Human Behavioral Medicine, Seoul, Republic of Korea; k Transdisciplinary Department of Medicine & Advanced Technology, Seoul National University Hospital, Seoul, Republic of Korea

**Keywords:** Bipolar II disorder, Major depressive disorder, Liquid chromatography-tandem mass spectrometry (LC-MS), Symptom severity, Proteomics

## Abstract

The proteomics data included in this article supplement the research article titled “Predictive protein markers for the severity of depression in mood disorders: A preliminary trans-diagnostic approach study (manuscript ID: JPSYCHIATRES-D-20-00437).” Plasma protein was analyzed using liquid chromatography-tandem mass spectrometry (LC-MS/MS). This data article included 370 plasma protein profiles expressed in patients with bipolar II disorder (BD-II) and major depressive disorder (MDD). The tables present the comparison of protein expressions between BD-II and MDD, and the relationship between the severity of the depressive symptoms and protein expression. In addition, details of results adjusting the use of each psychotropic medication (antipsychotics, mood stabilizers, and antidepressants) for 20 proteins that showed a significant relationship with the severity of the depressive symptom were presented in the table. Results of the bioinformatics analysis of proteins, which were significantly related to the severity of depressive symptom, are presented. The blood protein profiles and the results of the analyses presented in this data article provide detailed information on the proteins associated with mood disorders, and could be used as the basis for further mass spectrometry studies in psychiatric disorders.

## Specifications Table

**Table:** 

Subject	Biology
Specific subject area	Biochemistry, omics: proteomics,
Type of data	Tables
How data were acquired	Liquid chromatography-tandem mass spectrometric (LC-MS/MS) analysis, using a quadrupole orbitrap mass spectrometer (Thermo Scientific)
Data format	Analyzed data and filtered data
Parameters for data collection	Plasma sample was collected from 37 patients with bipolar II disorder and 34 with major depressive disorder.
Description of data collection	LC-MS/MS based proteomic profiling of plasma samples Hamilton Depression Rating Scale (HAM-D) 17 was used to assess depressive symptoms.
Data source location	Seoul National University Hospital, Seoul, Republic of Korea
Data accessibility	http://www.ebi.ac.uk/pride Project Name: Quantitative proteomic analysis of plasma in major mental disorders Project accession: PXD028841
Related research article	Hyunju Lee, Sang Jin Rhee, Jayoun Kim, Yunna Lee, Hyeyoung Kim, Junhee Lee, Kangeun Lee, Hyunsuk Shin, Hyeyoon Kim, Tae Young Lee, Minah Kim, Eun Young Kim, Se Hyun Kim, Yong Min Ahn, Jun Soo Kwon, Dohyun Han, Kyooseob Ha, Predictive protein markers for the severity of depression in mood disorders: A preliminary trans-diagnostic approach study, Journal of psychiatric research, Volume 142, October 2021, Pages 63–72 (https://doi.org/10.1016/j.jpsychires.2021.07.041 Get rights and content)

## Value of the Data

• Dataset represents protein profiles of plasma samples from patients with bipolar II disorder (BD-II) and major depressive disorder (MDD).
• For a total 370 protein profiles, statistical analysis results that compared the expression of each protein between the two groups (BD-II and MDD) were provided.
• Proteomics data, which showed the relationship with the severity of depression, are described in detail.
• Protein profiles of mood disorders can be used to serve as a valuable repository for various proteomics studies in the future.

## 1 Data Description

This data article includes the mass spectrometric profiles of 71 plasma samples in patients affected by mood disorder (37 with BD-II, 34 with MDD). Among 665 plasma proteins, which were quantified with at least two peptides, 370 proteins were selected after excluding proteins that were expressed in <30% of samples. For statistical analysis, a log2 transformation was conducted to adjust the skewed distribution of values, and missing values were inputted with a minimum value for each protein. To compare the differentially expressed proteins between BD-II and MDD, independent samples *t*-test was conducted, the results of which are described in Table S1. Results of Pearson's correlation analysis between the severity of depressive symptoms and each protein are described in Table S2. To adjust the effects of each drug use (antipsychotics, mood stabilizers, and antidepressants) that could influence the expression of proteins, multiple regression analysis was performed with significant proteins and the results are summarized in Table S3. False discovery rate (FDR) with the Benjamini-Hochberg correction was used for multiple comparisons. Bioinformatics analysis of proteins that significantly reflect the severity of symptoms was conducted using (DAVID) Bioinformatics Resources 6.8. The results of the gene ontology biological process analysis, cellular component analysis, and molecular function analysis are summarized in Table S4.

## 2 Experimental Design, Materials and Methods

### 2.1 Plasma sample preparation and the LC-MS/MS analysis

All detailed descriptions of the plasma sample preparation process and the liquid chromatography-tandem mass spectrometry (LC-MS/MS) analysis were described in the original article associated with this data paper (In press) and Kim et al. [1]. Plasma protein digestion was performed according to the previously reported method [2]. Briefly, 2 µl of plasma was mixed with digestion buffer (8 M urea, 5 mM TCEP, and 20 mM CAA in 0.1 M ABC). All resulting peptides were acidified with 10% trifluoroacetic acid and desalted using homemade C18-StageTips as previously described [3,4]. The desalted samples were dried with a vacuum dryer and stored at −80 °C until the LC-MS/MS analysis. The LC-MS/MS analysis was performed using Quadrupole Orbitrap mass spectrometers according to a modified version of a previously reported method [3,5].

### 2.2 Data processing for label free quantification

All data processing details were described in the article associated with this data paper (In press) and Kim et al. [1]. Raw MS files were processed with the MaxQuant software version 1.6.1.0 [6], and the Andromeda engine was used to match MS/MS spectra against the Human Uniprot protein sequence database (December 2014, 88,657 entries) and contaminant protein sequence [7]. Enzyme specificity was set to full tryptic digestion [5]. Peptides with a minimal length of six amino acids and up to two missed cleavages were considered, and the FDR was set to 1% at peptide, protein, and modification levels [5]. To maximize the quantification events across samples, matching between runs was performed using a previously reported in-house plasma spectral library [2]. The mass spectrometry proteomics data have been deposited to the ProteomeXchange Consortium via the PRIDE partner repository with the dataset identifier PXD028841 [8].

## Ethics Statement

Written informed consent was obtained from all patients before participation in the study. For patients younger than 18, informed consent was obtained from both the patient and their parents/guardians. The study protocol was approved by the Institutional Review Board of Seoul National University Hospital (IRB No. 1704-075-846) and was conducted in accordance with the principles of the Declaration of Helsinki.

## CRediT authorship contribution statement

**Hyunju Lee:** Data curation, Conceptualization, Formal analysis, Writing – original draft. **Sang Jin Rhee:** Conceptualization, Methodology, Writing – review & editing. **Jayoun Kim:** Formal analysis, Methodology, Writing – review & editing. **Yunna Lee:** Conceptualization, Methodology, Writing – review & editing. **Hyeyoung Kim:** Conceptualization, Methodology, Writing – review & editing. **Junhee Lee:** Conceptualization, Methodology. **Kangeun Lee:** Data curation. **Hyunsuk Shin:** Methodology, Resources, Investigation. **Hyeyoon Kim:** Methodology, Resources, Investigation. **Tae Young Lee:** Conceptualization, Methodology. **Minah Kim:** Conceptualization, Methodology. **Eun Young Kim:** Conceptualization, Methodology. **Se Hyun Kim:** Conceptualization, Methodology. **Yong Min Ahn:** Conceptualization, Methodology, Writing – review & editing. **Jun Soo Kwon:** Conceptualization, Methodology. **Dohyun Han:** Conceptualization, Resources, Investigation, Funding acquisition, Supervision. **Kyooseob Ha:** Conceptualization, Methodology, Resources, Funding acquisition, Supervision.

## Declaration of Competing Interest

The authors declare that they have no known competing financial interests or personal relationships which have or could be perceived to have influenced the work reported in this article.

## Appendix Supplementary Materials

Image, application 1

Image, application 2

Image, application 3

Image, application 4
